# Characterization of *Arcobacter* strains isolated from human stool samples: results from the prospective German prevalence study Arcopath

**DOI:** 10.1186/s13099-019-0344-3

**Published:** 2020-01-08

**Authors:** Vanessa Brückner, Ulrike Fiebiger, Ralf Ignatius, Johannes Friesen, Martin Eisenblätter, Marlies Höck, Thomas Alter, Stefan Bereswill, Markus M. Heimesaat, Greta Gölz

**Affiliations:** 1 Institute of Food Safety and Food Hygiene, Freie Universität Berlin, Berlin, Germany; 2 Institute of Microbiology, Infectious Diseases and Immunology, Charité - University Medicine Berlin, corporate member of Freie Universität Berlin, Humboldt-Universität zu Berlin, and Berlin Institute of Health, Berlin, Germany; 3 Labor 28, Berlin, Germany; 4 Synlab MVZ, Berlin, Germany; 5 Labor Limbach, Berlin, Germany

**Keywords:** Arcobacter, Human, Cytotoxicity, Virulence genes, Genotyping

## Abstract

**Background:**

*Arcobacter* constitute emerging food- and waterborne pathogens causing gastroenteritis in humans, but the underlying mechanisms are only incompletely understood. We therefore characterized *Arcobacter* isolates derived from human stool samples that had been collected during a prospective prevalence study in Germany in vitro. Thirty-six bacterial isolates belonging to the species *A. butzleri* (n = 24), *A. cryaerophilus* (n = 10) and *A. lanthieri* (n = 2) were genotyped by ERIC-PCR, the presence of 10 putative virulence genes was assessed and cytotoxic effects on the human intestinal cell line HT-29/B6 were analyzed applying the WST-assay.

**Results:**

Genotyping revealed high genetic diversity within the species *A. butzleri*, *A. cryaerophilus* and *A. lanthieri*. Both, *A. butzleri* and *A. lanthieri* encoded for a large number of putative virulence genes, while fewer genes were detectable in *A. cryaerophilus* isolates. Notably, the three cytolethal distending toxin (CDT) genes *cdtA*, *cdtB* and *cdtC* were abundant in both *A. lanthieri* isolates. Furthermore, all *A. butzleri* and *A. lanthieri*, but only one of the *A. cryaerophilus* isolates exerted cytotoxic effects.

**Conclusions:**

Our study provides evidence for the abundance of putative virulence genes in *Arcobacter* isolates and prominent cytotoxic effects of *A. butzleri* and *A. lanthieri *in vitro. The presence of *cdtA*, *cdtB*, *cdtC* in *A. lanthieri* points towards CDT secretion as potential mechanism underlying cytotoxicity as opposed to *A. butzleri*. However, the association of the *Arcobacter* virulence factors detected and human morbidity should be addressed in future studies.

## Background

*Arcobacter* constitute Gram-negative, motile bacilli belonging to the class of *Epsilonproteobacteria,* with 29 different species described so far [1]. In contrast to the genus *Campylobacter, Arcobacter* species are mostly aerotolerant and able to grow at temperatures below 30 °C [2]. *Arcobacter* have been isolated from different environmental sources, such as animals, food of animal origin, vegetables and surface water [3–5]. In animals, *Arcobacter* are mostly described as commensals but symptoms of infection like enteritis and mastitis have been reported [5]. In humans, *Arcobacter* infections are associated with gastroenteritis characterized by prolonged watery diarrhea and abdominal cramps, while single cases of bacteremia have been described [3, 6, 7]. Since 2002, the *Arcobacter* species *A. butzleri* and *A. cryaerophilus* have been classified as “serious hazard to human health” by the International Commission on Microbiological Specification for Foods [8]. However, both, the prevalence and significance of *Arcobacter* infections in humans are most likely underestimated, given the lack of standardized isolation procedures.

When assessing the potential pathomechanisms underlying *Arcobacter* induced disease, several studies revealed adhesive, invasive and cytotoxic properties of *Arcobacter* spp., with slightly different conclusions depending on the strains investigated, cell lines included and methods applied [3, 5, 9–13]. *A. butzleri* have been shown to compromize the barrier function in epithelial monolayers of HT-29/B6 cells in vitro, a mechanism, which might be responsible for the diarrhea induced by *Arcobacter* spp.[14]. However, the relevant virulence factors of *Arcobacter* spp. are yet to be identified. Within the whole genome sequence of *A. butzleri* RM4018, the ten putative virulence factors *cadF*, *cj1349*, *ciaB*, *pldA*, *tlyA*, *mviN*, *hecA*, *hecB*, *irgA* and *iroE* have been determined, known to be involved in the pathogenicity of other bacteria [15]. So far, no correlation between the occurrence of the respective putative virulence genes in *Arcobacter* and their pathogenic potential could be unraveled. Furthermore, no toxin, which might be responsible for the cytotoxic effects reported for *A. butzleri*, has been identified yet. In contrast to *A. butzleri* and *A. cryaerophilus*, the cytolethal distending toxin (CDT) encoding genes *cdtABC* have been detected in several *A. lanthieri* isolates [16].

In a very recent prospective human prevalence study in Germany, we surveyed almost 4700 stool samples for the prevalence of *Arcobacter*. The stool samples had been collected at three microbiological diagnostic laboratories in Berlin, Germany, and were submitted for the detection of bacterial enteropathogens. Among the detected *Arcobacter*, *A. butzleri* was the most prevalent species, followed by *A. cryaerophilus* and *A. lanthieri* (GUTP-D-19-00199).

The aim of present study was to characterize the 36 *Arcobacter* isolates derived from the above mentioned human prevalence study in terms of their (i) genotype, (ii) presence of virulence genes and (iii) cytotoxic potential in vitro.

## Results

### Genotyping of *Arcobacter* isolates

**Fig. 1:**
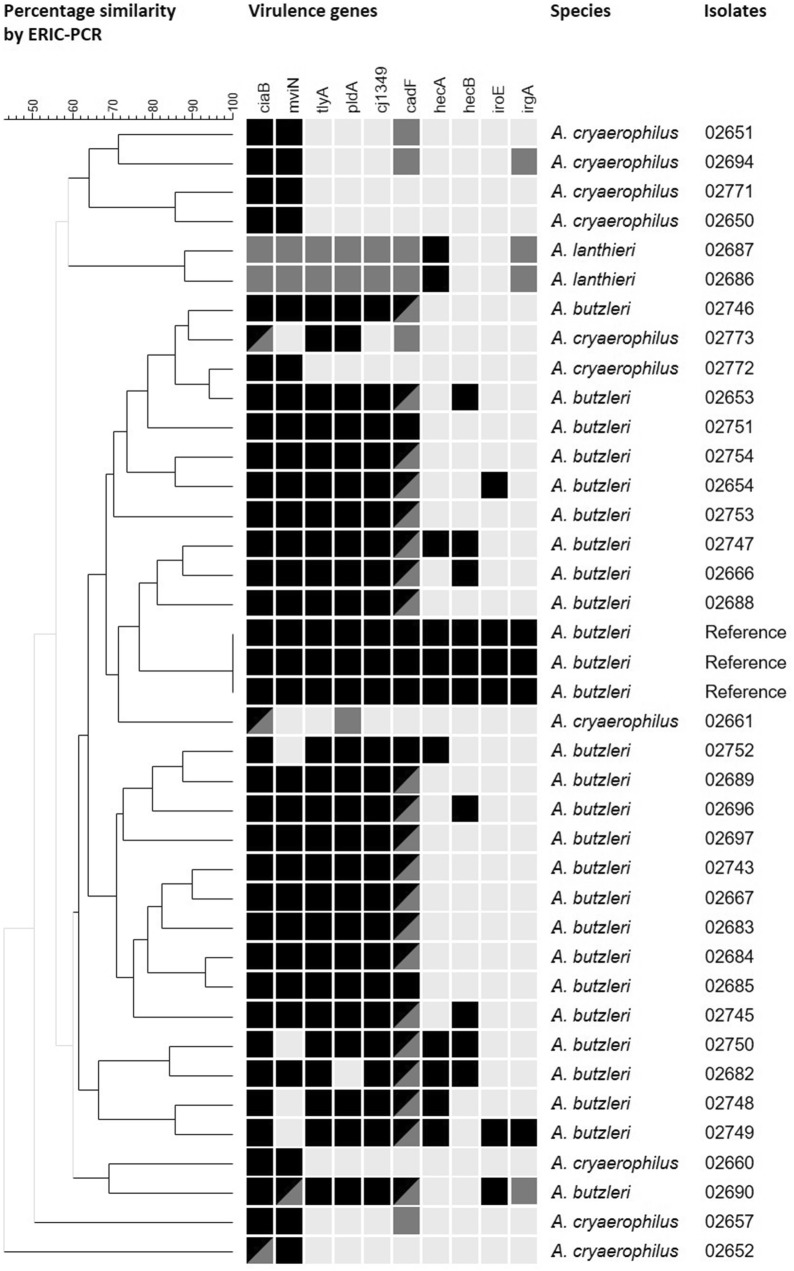
Dendrogram based on ERIC-PCR assay using Dice similarity coefficient and UPGMA method and virulence gene pattern of *Arcobacter* spp. isolated from human stool samples. Black: genes detected by primers designed based on *A. butzleri* sequences (Douidah et al. [25], Karadas et al. [11], Whiteduck-Leveillee et al. [40]; pale grey: genes not detected by both PCR; dark grey: genes detected by primers designed based on *A. lanthieri* sequences (Zambri et al. [16]). *A. butzleri* (CCUG 30485) was included as reference strain

Enterobacterial repetitive intergenic consensus (ERIC) sequences were detected in all 36 *Arcobacter* isolates, and different fragment patterns were generated consisting of 5 to 15 fragments ranging from approximately 100–1000 bp in length. For analysis of fragment patterns, the similarities were calculated using Dice coefficient followed by the Unweighted Pair Group Method with Arithmetic mean (UPGMA) to generate a dendrogram, showing the level of genetic similarity (Fig. 1). Dendrogram analyses revealed a high genetic diversity, particularly among the *A. cryaerophilus* isolates. Only the *A. butzleri* isolates were clustering in one large group with 60% similarity. This cluster also included three independent replicates of the human *A. butzleri* reference strain (CCUG30485) with identical fragment pattern, indicating the reproducibility of the method applied. Interestingly, also three *A. cryaerophilus* isolates were included in this cluster. In fact, no species-specific cluster could be identified for isolates of *A. cryaerophilus*, while both *A. lanthieri* isolates were clustering at a high similarity level (86%).

### Abundance of putative virulence genes

Additionally, we analyzed the presence of the ten putative virulence genes described for *A. butzleri* [15]. A majority of these putative virulence genes were detected in *A. butzleri*, while considerably fewer genes were assessed in *A. cryaerophilus* (Fig. 1). In fact, the genes *ciaB, cj1349, cadF,* and *tlyA* were present in all *A. butzleri* (n = 24) isolates, whereas *pldA*, *mviN*, *hecB,* and *hecA* were detectable in 96%, 83%, 29%, and 25% of the isolates, respectively. In contrast, the genes *iroE* and *irgA* were less frequently detected, namely in 12% (n = 3) and 8% (n = 2) of the isolates, respectively. Among the ten *A. cryaerophilus* strains, *ciaB* was present in all isolates, while the *mviN*, *cadF* and *pldA* genes were detectable in eight, four and two isolates, respectively. Two further genes, namely *tlyA* and *irgA,* were each detected in a single *A. cryaerophilus* isolate.

When using *A. butzleri*-specific PCR-primers, only the presence of *hecB* could be assessed in both *A. lanthieri* strains, whereas *ciaB*, *mviN*, *cadF*, *pldA*, *tlyA,* and *irgA* were only detected by using the *A. lanthieri*-specific primers. Furthermore, the three genes encoding for the CDT, i.e., *cdtA*, *cdtB,* and *cdtC,* were present in both *A. lanthieri* strains (data not shown).

### Cytotoxic effects in vitro

**Fig. 2:**
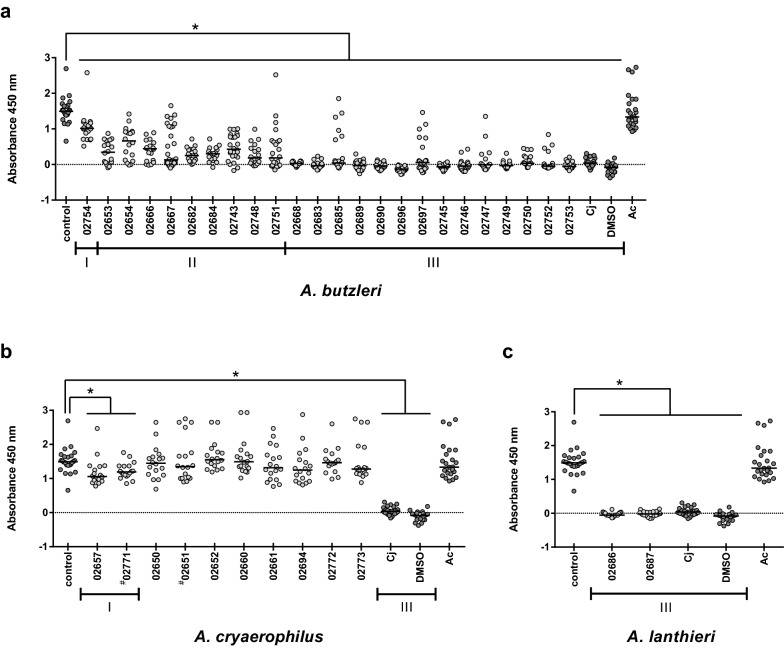
Viability of HT-29/B6 cells after inoculation with *Arcobacter* isolates. HT-29/B6 cells, differentiated for 7 days, were inoculated with *A. butzleri* (**a**), *A. cryaerophilus* (**b**) and *A. lanthieri* (**c**) isolates (MOI 100, except for isolates 02651 and 02771 that were incubated at MOI 50), and cytotoxicity was measured after 48 h incubation by WST-1 assay. At least three independent experiments were performed with six replicates each. Cells treated with medium only or with *A. cryaerophilus* ILSH 02659 (Ac) were included as negative control, and dimethyl sulfoxide (DMSO) and *Campylobacter jejuni* 81–176 (Cj) as positive controls. The isolates were arbitrarily classified in three groups due to the level of toxicity, i.e., isolates of low (group I), moderate (group II) and high cytotoxicity (group III) with 20–49%, 50–94% and at least 95% reduction of absorbance as compared to the negative control, respectively. ^#^ inoculation at MOI 50; * p < 0.05 (Mann–Whitney U-test) as compared to control

Finally, we assessed potential cytotoxic effects of the isolated *Arcobacter* isolates in vitro by using the human intestinal cell line HT-29/B6. HT-29/B6 cells were incubated for 48 h at 37 °C with the *Arcobacter* isolates (MOI of 100). Cytotoxicity was determined by measuring the residual viability of HT-29/B6 cells in the colorimetric WST-assay. Inoculation with the human isolate *A. cryaerophilus* (ILSH 02659), which was included as negative control, revealed no significant changes in absorbance compared to uninfected media control (10%) indicating that most cells remained metabolically active. Inoculation with all *A. butzleri* strains, both outpatient and in-clinic isolates, however, resulted in a significant reduction of absorbance compared to media control, indicative for significant cytotoxic effects on HT-29/B6 cells (Fig. 2a). Fourteen isolates were identified as high cytotoxic strains (Group III), causing a decrease in absorbance by at least 95% compared to control, which was comparable to the reductions induced by the positive controls, *C. jejuni* 81–176 (97%) and DMSO (105%), respectively. Absorbances measured after inoculation with another nine isolates were significantly reduced by 50% to 95% compared to control, indicative for moderate cytotoxicity (Group II). In contrast to all other *A. butzleri* isolates, only strain 02754 induced a smaller decrease in absorbance by 32%, demonstrating relatively low cytotoxic activity (Group I).

In contrast to *A. butzleri*, the majority of the investigated *A. cryaerophilus* isolates (8 out of 10) did not induce cytotoxic effects on HT-29/B6 cells. Due to limited bacterial growth, however, the inoculation with isolates 02651 and 02771 had to be performed at MOI 50 instead of MOI 100; hence, respective results should be interpreted with caution. The inoculation with these isolates resulted in absorbances that were not significantly different from media control (Fig. 2b). Only isolates 02657 and 02771 exerted a significant reduction of absorbance by 29% and 20%, respectively, as compared to control, pointing towards some cytotoxic activity (Group I).

Both *A. lanthieri* isolates induced high cytotoxicity (Group III) in HT-29/B6 cells, yielding a reduction of absorbance by at least 96%, that was comparable to *C. jejuni* and DMSO control (Fig. 2c).

## Discussion

### Genotyping

Various reports using ERIC-PCR to unravel the genetic diversity of *Arcobacter* have described a large heterogeneity among this bacterial genus [17–19]. Likewise, Mandisodza et al. [7] described 12 different pulsotypes using pulsed field gel electrophoresis among 7 *A. butzleri* and 5 *A. cryaerophilus* strains from human diarrheal cases. These findings are in agreement with our data; in fact, we here observed a unique genotype for each isolate. While dendrogram analysis revealed species-specific cluster for most *A. butzleri* and both *A. lanthieri* strains, *A. cryaerophilus* isolates were widely distributed within the dendrogram. Based on genomic comparisons, a recently published study has suggested to subdivide the species *A. cryaerophilus* into four separate genomovars [20] which might explain the high heterogeneity of the *A. cryaerophilus* detected in our survey.

Houf et al. [21] have reported 91 genotypes of *A. butzleri* out of 182 isolates and 40 genotypes of *A. cryaerophilus* out of 42 isolates obtained from poultry products, and 91.2% of the isolates obtained from sewage effluent showed different fragment patterns [22]. Importantly, the different genotypes are not associated with the source of the isolates [23]. However, Sekhar et al. [24] have recently shown genetic similarity of isolates from animal and human origin determined by rep-PCR cluster analysis, indicating the possibility of zoonotic transmission. Further investigations are necessary to identify distinct sources of infection and transmission routes of *Arcobacter*.

### Virulence genes

Our study revealed the abundance of ten putative virulence genes with homologies to virulence factors of other bacteria, particularly *C. jejuni*. In agreement with our data, six of these genes, i.e., *ciaB, cj1349, cadF*, *tlyA*, *pldA,* and *mviN*, have been detected most frequently in *A. butzleri* strains isolated from various sources, with prevalences ranging from 66 to 100% [11, 23, 25–31]. We found *hecB, hecA* and *irgA* less frequently and at lower rates as compared to other reports [11, 23, 25, 27, 29, 31, 32]. The least frequently detected gene in our survey was *irgA,* which is in line with other studies [11, 26, 27, 33], although *irgA* rates of 25–46% have also been reported [23, 25, 29, 31]. The presence of *iroE* has rarely been investigated with prevalences of 17–60% [11, 23, 26, 27], which is slightly higher than the detected 13% in our study. Nevertheless, considering all studies published *hecB*, *hecA*, *irgA,* and *iroE* appear to be less common in *A. butzleri*. In summary, in none of the *A. butzleri* isolates, we detected all of the putative virulence genes investigated. This difference to other studies describing 10–23% of the strains derived from different sources to possess all of the analyzed genes may be due to the different sources of the bacterial isolates [11, 23, 26, 27], since we investigated exclusively human isolates.

Overall, we identified less virulence genes in *A. cryaerophilus* than in *A. butzleri*. While *ciaB* was detected in all *A. cryaerophilus* isolates, the genes *mviN*, *cadF,* and *pldA* were found in 80%, 40%, and 20% of isolates, respectively, and *tlyA* as well as *irgA* in 10% of isolates. The *ciaB* and *mviN* genes have previously been reported to be more abundant than other putative virulence genes in *A. cryaerophilus* [11, 23, 25, 29, 31], and also our data regarding the detection of *tlyA* and *pldA* are in line with these studies*.* Furthermore, we detected *cadF* in 40% of isolates, which is in concordance with other studies reporting the presence of *cadF* in 10–62% of isolates [25, 26, 28–30]. While we were not able to detect any further virulence genes in *A. cryaerophilus*, other studies have reported the presence of *cj1349* in 20–77% of isolates [25, 26, 28–30], and also the genes *hecB, hecA,* and *irgA* were identified more often than in our study [25]. These differences might be due to the genomic heterogeneity in primer binding sequences.

When analyzing the virulence genes of *A. lanthieri* the use of *A. butzleri* specific primers allowed the detection of only *hecB* in both *A. lanthieri* isolates, whereas applying the species-specific primer revealed six additional genes*.* Based on this, *A. lanthieri* displays a similar virulence gene pattern as *A. butzleri*. We also detected more virulence genes in *A. cryaerophilus* by the primers designed on the base of *A. lanthieri* than of *A. butzleri* sequences. This underlines the need of genus-specific primers for the detection of virulence genes in *Arcobacter*, which needs to be addressed in future studies. Furthermore, we detected the *cdtA*, *cdtB,* and *cdtC* genes encoding for CDT in both *A. lanthieri* isolates, which are also expressed by several *C. jejuni* strains [34] and likely contribute to their pathogenicity [35, 36].

### Cytotoxic effects

In addition, we addressed cytotoxic properties of the isolates in vitro by using the WST-1 assay. Our results indicate moderate to high levels of cytotoxicity for most *A. butzleri* isolates, which is in agreement with a previous study where cytotoxicity also was determined by measuring the activity of mitochondrial dehydrogenases [10]. In contrast, other studies assessed the cytotoxic effects by microscopical examination [9, 37, 38], and observed cytotoxic effects of broth culture filtrates of *A. butzleri* on Vero and CHO cells. Besides secretion of an enterotoxin, the production of a vacuolizing toxin-like factor has been postulated [13].

Cytotoxicity of *A. cryaerophilus* has rather rarely been demonstrated [13, 39]. This discrepancy to our findings might be due to the different methods, cell lines or strains used. Furthermore, our study is the first one assessing cytotoxic activity of *A. lanthieri* isolates derived from human stool specimens, and both isolates exhibited a high degree of cytotoxicity. Still, this finding needs to be confirmed by analyzing larger number of isolates.

It yet remains unclear, which gene(s) might be involved in exerting the cytotoxic effects. In *A. butzleri*, however, a toxin different to CDT is assumed to be encoded [34], since genome sequencing of the reference strain *A. butzleri* RM1408 [15] and also of selected *A. butzleri* strains investigated by us revealed the absence of CDT genes. This is further supported by cytotoxicity of CDT-negative *Arcobacter* [39]. Notably, we here detected all three CDT toxin genes in both *A. lanthieri* isolates, which is in concordance with results from Zambri et al. [16], who detected these in 8 out of 11 *A. lanthieri* isolates from various environmental and fecal sources. Thus, while CDT may likely be involved in *A. lanthieri*-induced cytotoxicity, other factors might contribute to cytotoxic effects of *A. butzleri*. For example, the two putative virulence genes *cj1349* and *tlyA* were present in all *A. butzleri* and *A. lanthieri* isolates, displaying cytotoxic effects on HT-29/B6 cells. In other bacterial species, *cj1349* has been shown to be associated with the adhesion to intestinal cells and *tlyA* with hemolysis of erythrocytes. In a recent study of Karadas et al. [10] six *A. butzleri* isolates, all encoding for the adhesion gene *cj1349*, displayed differently prominent adhesive phenotypes, whereas no correlation between the adhesive phenotypes and respective amino acid sequences could be shown, however. Furthermore, two isolates exerting only low or no adhesive capacities at all were even highly cytotoxic [11]. Therefore, based upon our obtained results it appears rather difficult to draw definite conclusions whether *cj1349* is involved in the capacity of *Arcobacter* spp. exerting cytotoxicity, whereas *tlyA* might represent a putative virulence factor, which needs, however, to be further investigated in this regard.

## Conclusions

In this study, characterization of human *Arcobacter* isolates revealed an abundance of putative virulence genes and prominent in vitro cytotoxic effects of *A. butzleri* and *A. lanthieri*. Furthermore, the presence of *cdtA*, *cdtB*, and *cdtC* in *A. lanthieri,* but not in *A. butzleri* indicates that CDT production might contribute to cytotoxicity exerted by *A. lanthieri.* Further studies are warranted for further in-depth evaluation of the role of *Arcobacter* in human disease.

## Methods

### Bacterial strains and culture conditions

A total of 36 human *Arcobacter* isolates (24 *A. butzleri*, 10 *A. cryaerophilus* and 2 *A. lanthieri*) and the reference strain *A. butzleri* (CCUG 30485) were included. The 36 human isolates were collected during a previous prospective *Arcobacter* prevalence study in Germany by using selective enrichment media, and species verified by multiplex PCR and *rpoB* sequencing (GUTP-D-19-00199). All incubation steps were performed at 30 °C in Brucella broth (BB; BD, Heidelberg, Germany) or on Mueller–Hinton agar plates (Oxoid) supplemented with 5% sheep blood (MHB) under microaerobic conditions unless stated differently.

### Genotyping by ERIC-PCR

For evaluating genetic diversity the identified isolates were characterized by ERIC-PCR as previously described [21]. For amplification of the intergenic sequences between the ERIC-sequences the ERIC motifs 1R and 2 were used (Table 1). The PCR reaction mixture contained 1 × PCR buffer (Qiagen, Venlo, Netherlands), 4 mM MgCl_2_ (Qiagen), 0.2 mM of each deoxynucleoside triphosphate (dNTP; Thermo Fisher Scientific, Waltham, USA), 2.5 U *Taq* polymerase (Qiagen), 0.5 µM of each primer and 1 µl template DNA in a total reaction volume of 25 µl. PCR samples were subjected to an initial denaturation at 94 °C for 5 min, followed by 40 amplification cycles, consisting of denaturation at 94 °C for 1 min, annealing at 25 °C for 1 min and elongation at 72 °C for 2 min, and subsequently 5 min at 72 °C for final extension. Amplified products were separated using gel electrophoresis and visualized under UV light by GRgreen staining. Analyses of respective fragment patterns were performed by using BioNumerics version 7.1 (Applied Maths, Sint- Martens-Latem, Belgium). The similarities between profiles were calculated using Dice coefficient, and the UPGMA method was used for cluster analysis and to generate dendrograms.

**Table 1:** List of primers used in this study

Primer	Sequence (5′-3′)	Product size (bp)	References
Genotyping			
ERIC 1R	ATG TAA GCT CCT GGG GAT TCA C		Houf et al. [21]
ERIC 2	AAG TAA GTG ACT GGG GTG AGC G		Houf et al. [21]
Detection of putative virulence genes			
cadF-F	TTA CTC CTA CAC CGT AGT	283	Douidah et al. [25]
cadF-R	AAA CTA TGC TAA CGC TGG TT		Douidah et al. [25]
irgA-F	TGC AGA GGA TGC TTG GAG CGT AAC T	437	Whiteduck-Leveillee et al. [40]
irgA-R	GTA TAA CCC CAT TGA TGA GGA GCA		Whiteduck-Leveillee et al. [40]
hecA-F	GTG GAA GTA CAA CGA TAG CAG GCT C	537	Whiteduck-Leveillee et al. [40]
hecA-R	GTC TGT TTT AGT TGC TCT GCA GTC		Whiteduck-Leveillee et al. [40]
hecB-F	CTA AAC TCT ACA AAT CGT GC	528	Whiteduck-Leveillee et al. [40]
hecB-R	CTT TTG AGT GTT GAC CTC		Whiteduck-Leveillee et al. [40]
pldA-F	TTG ACG AGA CAA TAA GTG CAG C	293	Whiteduck-Leveillee et al. [40]
pldA-R	CGT CTT TAT CTT TGC TTT CAG GGA		Whiteduck-Leveillee et al. [40]
ciaB-F	TGG GCA GAT GTG GAT AGA GCT TGG A	284	Whiteduck-Leveillee et al. [40]
ciaB-R	TAG TGC TGG TCG TCC CAC ATA AAG		Whiteduck-Leveillee et al. [40]
cj1349-F	CCA GAA ATC ACT GGC TTT TGA G	659	Whiteduck-Leveillee et al. [40]
cj1349-R	GGG CAT AAG TTA GAT GAG GTT CC		Whiteduck-Leveillee et al. [40]
tlyA-F	CAA AGT CGA AAC AAA GCG ACT G	230	Whiteduck-Leveillee et al. [40]
tlyA-R	TCC ACC AGT GCT ACT TCC TAT A		Whiteduck-Leveillee et al. [40]
mviN-F	TGC ACT TGT TGC AAA ACG GTG	294	Whiteduck-Leveillee et al. [40]
mviN-R	TGC TGA TGG AGC TTT TAC GCA AGC		Whiteduck-Leveillee et al. [40]
iroE-F	AAT GGC TAT GAT GTT GTT TAC	415	Karadas et al. [11]
iroE-R	TTG CTG CTA TGA AGT TTT		Karadas et al. [11]
Detection of putative virulence genes with *A. lanthieri* specific primers			
AL_cdtB F	GCA AAA GGT GAT TGG GCT CC	303	Zambri et al. [16]
AL_cdtB R	TCC TCC AGC TCC TTG AAC AC		Zambri et al. [16]
AL_cadF F	TCC AAC TCC AGT TGC TGC TC	243	Zambri et al. [16]
AL_cadF R	TGT CCT TCG ATG TCA GCT TTC		Zambri et al. [16]
AL_irgA F	AGA GCT GTT GGT TGG GAT GG	186	Zambri et al. [16]
AL_irgA R	TGC ATT TGC TCT TGT AGG GT		Zambri et al. [16]
AL_cdtC F	GAT GAA TCC ACC AGA AAT AGA G	196	Zambri et al. [16]
AL_cdtC R	TTT GGG ATC AAG AGT ATA AAG TTC		Zambri et al. [16]
AL_pldA F	TGC TCC ATT TAG AGA AAC TAA C	132	Zambri et al. [16]
AL_pldA R	GAA CGA GAT TCT TCA CCA TCT T		Zambri et al. [16]
AL_cdtA F	CAG GAA TAG ATC TCG CTA CAA ATG	220	Zambri et al. [16]
AL_cdtA R	TTT GGT AGA AGA GGA AGT TCA TTG		Zambri et al. [16]
AL_mviN F	ACC TTT GGT TCT TCA ACT TTA C	170	Zambri et al. [16]
AL_mviN R	CGT GCT ACC ATA GGA AAT AGG		Zambri et al. [16]
AL_ciaB F	GAT AGA TGC TAT TCT GCT CTT G	207	Zambri et al. [16]
AL_ciaB R	ATC TTC ACT AAA TGC TAC TAT T		Zambri et al. [16]
AL_tlyA F	GAC ATT GTA ACA TGT GAT GTA TCT T	125	Zambri et al. [16]
AL_tlyA R	TTT ACA TTT GTT CCC ACT TCA AA		Zambri et al. [16]

### Presence of virulence associated genes

All primers used are listed in Table 1. PCR protocols for partial amplification of *ciaB, mviN, pldA, tlyA, irgA, hecA, hecB* and *cj1349* were used according to Whiteduck-Leveillee et al. [40]. Briefly, 25 µl PCR-mixture contained 2 µl template DNA, 1 × PCR buffer, 1.5 mM MgCl_2_, 0.2 mM of each dNTP, 0.5 U *Taq* polymerase and 0.1 µM of corresponding primers. Reaction conditions were 95 °C for 4 min followed by 30 cycles of 95 °C for 30 s, 56 °C for 45 s and 72 °C for 45 s and ended with a final extension step at 72 °C for 5 min. Partial amplification of *iroE* and *cadF* was carried out according to the protocol of Karadas et al. [11]. The PCR-mixture contained the same composition as described above except for the primers being at 1 µM. Reaction conditions were 95 °C for 4 min followed by 30 cycles of 95 °C for 30 s, 50 °C for 30 s and 72 °C for 30 s and ended with a final extension step at 72 °C for 5 min.

For analysis of *A. lanthieri* additional primers and a protocol described by Zambri et al. [16] were used for the detection of *ciaB*, *mviN*, *cadF*, *pldA*, *tlyA*, *irgA*, *cdtA*, *cdtB* and *cdtC*. Briefly, 25 µl PCR-mixture contained 2 µl template DNA, 1 × PCR buffer, 1.5 mM MgCl_2_, 0.2 mM of each dNTP, 0.5 U *Taq* polymerase and different concentrations of corresponding primers (0.4 µM of each AL_cdtB, AL_cadF, AL_irgA, and AL_mviN; 0.3 µM of each AL_cdtC and AL_cdtA; 0.2 µM of each AL_ciaB; 0.1 µM of each AL_pldA and AL_tlyA). Reaction conditions were 94 °C for 2 min followed by 35 cycles of 95 °C for 30 s, primer specific annealing temperatures for 45 s (56 °C for *cdtB, cadF, irgA*; 57 °C for *cdtC, pldA*; 55 °C for *cdtA, mviN*; 60 °C for *ciaB, tlyA*) and 72 °C for 30 s and ended with a final extension step at 72 °C for 5 min. Amplified products were separated using gel electrophoresis and visualized under UV light by GRgreen staining.

### Cell culture

The human colon adenocarcinoma cell line HT-29/B6 [41] was grown in a 75 cm^2^ tissue culture flask (Sarstedt, Nümbrecht, Germany) containing RPMI1640 medium (Lonza Bioscience, Cologne, Germany) supplemented with 10% fetal bovine serum (FBS) (Lonza Bioscience) and 10 µg/ml gentamicin (Biochrom, Berlin, Germany) at 37 °C in a 5% CO_2_ humidified atmosphere.

### Cytotoxicity analysis

The WST-assay (as described by Karadas et al. [10]) was used to assess cytotoxic effects of *Arcobacter* isolates on the human intestinal cell line, HT-29/B6. This colorimetric assay is based on enzymatic conversion of tetrazolium salt WST-1 (4-[3-(4-Iodophenyl)-2-(4-nitro-phenyl)-2*H*-5-tetrazolio]-1,3-benzene disulfonate) to formazan by cellular mitochondrial dehydrogenases, present in viable cells, resulting in a color change from red to orange. The measured absorbance directly correlates with the number of metabolically active cells and therefore, also reflects cytotoxic effects indicated by a decrease in cell proliferation.

HT-29/B6 cells were seeded in 96-well-plates (Sarstedt) at a density of 3 × 10^4^ cells/well (100 µl each well). After differentiation for 7 days at 37 °C in a humidified incubator with 5% CO_2_, cells were washed with phosphate-buffered saline (PBS, Sigma-Aldrich) and antibiotic-free medium was added prior to *Arcobacter-*treatment.

*Arcobacter* isolates were precultured overnight in BB. The precultures were diluted 1:100 in 10 ml BB and further incubated overnight. The overnight cultures were centrifuged at 5000×*g* for 10 min and pellets resuspended in 1 ml PBS resulting in approximately 1 × 10^8^ CFU in the inoculum volume of 50 µl. To receive similar concentrations for *A. cryaerophilus* isolates, due to slower growth, three overnight cultures of each isolate were prepared in BB and incubated for 48 h. After centrifugation of pooled cultures, pellets were resuspended in 600 µl PBS. *C. jejuni* (81–176) was used as reference strain and processed as described but at 37 °C.

Prepared bacterial inocula (50 µl) were added to HT-29/B6 cells in 96-well plate with a multiplicity of infection (MOI) of 100 and incubated for 48 h at 37 °C with 5% CO_2_. As negative controls, cells were treated with medium only or with *A. cryaerophilus* (ILSH 02659), a strain without cytotoxic effect in this assay. As positive controls, dimethyl sulfoxide (DMSO) and *C. jejuni* (81–176) were used. The WST-1 cell proliferation assay kit (Roche Applied Science, Mannheim, Germany) was used according to the manufacturer’s instructions. Briefly, the wells were washed once with PBS before adding 110 µl WST-1-reagent to each well. After 1 h incubation (37 °C, 5% CO_2_) 100 µl of the supernatant were transferred to a new 96-well plate prior to measuring the absorbance of the formazan product at 450 nm using a microplate reader (FLUOstar OPTIMA; BMG Labtech, Ortenberg, Germany). The obtained data were corrected by *subtracting* the reagent *blank* from each of the other determined *values.* At least three independent experiments were performed with six replicates each. The level of toxicity was arbitrarily classified, i.e., high, moderate and low cytotoxicity characterized by at least 95%, 50–94% and 20–49% reduction of absorbance as compared to uninfected media control, respectively.

### Statistical analyses

For each isolate at least three independent experiments were performed with six replicates each, and data analyzed by using GraphPad Prism (version 5.04; GraphPad Software, Inc., La Jolla, US). The nonparametric two-tailed Mann–Whitney U Test was used to calculate significant differences in cytotoxic effects of *Arcobacter* isolates. Two-sided probability (p) values ≤ 0.05 were considered significant.

## Data Availability

All data generated or analysed during this study are included in this published article.
